# Supplementary material for: Analysis of PPARγ Signaling Activity in Psoriasis
Source: Int J Mol Sci. 2021 Aug 10;22(16):8603. doi: 10.3390/ijms22168603 (PMC8395241; doi:10.3390/ijms22168603)
Supplement: Supplementary file 1 [file ijms-22-08603-s001.zip › Supplemental materials_Analysis of PPARg signaling activity in psoriasis/Pathway models/Models images and html files/PPARG expressed in cells assotiated with psoriasis/226705.html]

sebocyte


# CellType sebocyte

|  |  |
| --- | --- |
| URN | urn:agi-ncimcelltype:C1710037 |
| Total Entities | 0 |
| Connectivity | 386 |
| Name | sebocyte |

---

|  |  |
| --- | --- |
| Pathway | EGFR -> Expression Targets in Skin |
|  | PPARG expressed in cells assotiated with psoriasis |
|  | Overview. Diseases of the skin (chapter 11) |

---

|  |  |
| --- | --- |
| MedScan ID | 10004086 |

---

|  |  |
| --- | --- |
| Alias | sebaceous tissue cell |
|  | sebaceous tissue cells |
|  | sebocytes |
|  | sebocyte |
|  | sebaceous epithelial cell |
|  | sebaceous epithelial cells |
|  | sebaceous gland cell |
|  | sebaceous cells |
|  | sebaceous gland cells |
|  | sebaceous cell |

---

|  |  |
| --- | --- |
| NCIm ID | C1710037 |

---
